# Renal Expression of Annexin A1 Is Associated With the Severity of Renal Injury in Antineutrophil Cytoplasmic Autoantibody-Associated Vasculitis

**DOI:** 10.3389/fmed.2022.769813

**Published:** 2022-06-17

**Authors:** Rui-Xue Wang, Liang Wu, Su-Fang Chen, Zhi-Ying Li, Ming-Hui Zhao, Min Chen

**Affiliations:** ^1^Renal Division, Department of Medicine, Peking University First Hospital, Beijing, China; ^2^Institute of Nephrology, Peking University, Beijing, China; ^3^Key Laboratory of Renal Disease, Ministry of Health of China, Beijing, China; ^4^Key Laboratory of Chronic Kidney Disease Prevention and Treatment (Peking University), Ministry of Education, Beijing, China; ^5^Research Units of Diagnosis and Treatment of Immune-Mediated Kidney Diseases, Chinese Academy of Medical Sciences, Beijing, China; ^6^Peking-Tsinghua Center for Life Sciences, Beijing, China

**Keywords:** annexin A1, ANCA, vasculitis, renal injury, disease severity

## Abstract

**Background:**

Increasing studies demonstrated the importance of activation of neutrophils in the pathogenesis of antineutrophil cytoplasmic autoantibody (ANCA)-associated vasculitis (AAV). Previous studies showed that annexin A1 (ANXA1) inhibited the recruitment, transendothelial migration and respiratory burst of neutrophils and induced apoptosis of neutrophils. The current study aimed to investigate the plasma and renal levels of ANXA1 as well as their association with the disease severity in AAV patients.

**Methods:**

Thirty-one AAV patients in active stage and 35 AAV patients in remission stage were recruited. The expression of ANXA1 in renal specimens was assessed by immunohistochemistry. The co-localization of ANXA1 with renal intrinsic and infiltrating cells was detected by double immunofluorescence. The plasma levels of ANXA1 were determined by ELISA. The association of plasma and renal levels of ANXA1 with clinicopathological parameters was further analyzed.

**Results:**

Plasma levels of ANXA1 were significantly higher in active AAV patients than those in AAV patients in remission as well as healthy controls. The renal expression of ANXA1 was significantly higher in active AAV patients than in healthy controls and disease controls. Double immunofluorescence assay showed that ANXA1 was expressed in glomerular endothelial cells, mesangial cells, podocytes, proximal tubular epithelial cells, neutrophils, monocytes/macrophages and T cells in AAV patients. The mean optical density of ANXA1 in glomeruli was correlated with serum creatinine levels (r = −0.491, *P* = 0.005) and eGFR (*r* = 0.492, *P* = 0.005) at renal biopsy and the proportion of crescents (*r* = −0.423, *P* = 0.018) in renal specimens of AAV patients. The expression of ANXA1 in glomeruli of AAV patients achieving complete renal recovery was significantly higher than those achieving partial renal recovery.

**Conclusion:**

In AAV patients, the renal expression of ANXA1 was associated with the severity of renal injury.

## Introduction

Anti-neutrophil cytoplasmic antibody (ANCA)-associated vasculitis (AAV) comprises microscopic polyangiitis (MPA), granulomatosis with polyangiitis (GPA) and eosinophilic granulomatosis with polyangiitis (EGPA), characterized by necrotizing inflammation of the small blood vessels (1). ANCAs against myeloperoxidase (MPO) or proteinase 3 (PR3) are the serological markers of AAV (2, 3). Glucocorticoids combined with cyclophosphamide (CTX) or rituximab in induction therapy and azathioprine (AZA) or rituximab in maintenance therapy was the most commonly used regimen for the treatment of AAV (4, 5).

Although the pathogenesis of AAV has not been fully elucidated, increasing studies have emphasized the importance of ANCA, neutrophil activation and the complement system [reviewed in (6)]. ANCA could induce neutrophils to undergo respiratory burst and degranulation, which resulted in release of free oxygen radicals and various proteases and caused further lesions of vasculitis (2, 7–9).

As a member of the annexin superfamily, annexin A1 (ANXA1) is involved in a variety of cellular biology processes, including cell proliferation, differentiation and apoptosis [reviewed in (10)]. Specifically, ANXA1 peptide can inhibit the recruitment, transendothelial migration and respiratory burst of neutrophils (11–13). ANXA1 can induce neutrophils apoptosis *in vitro* and *in vivo* (14, 15). On the other hand, as a downstream effector of glucocorticoids, ANXA1 contributes to the anti-inflammatory effect of glucocorticoids on the innate immune system (16).

It was demonstrated in animal studies that ANXA1 could alleviate the disease severity of various autoimmune diseases, including inflammatory bowel disease (IBD) and rheumatoid arthritis (RA) (17, 18). Besides, circulating levels of ANXA1 in patients with relapsing/remitting multiple sclerosis inversely correlated with the disease severity (19). A recent proteomic analysis of neutrophils in GPA patients revealed that a group of dysregulated proteins, including ANXA1, were associated with apoptosis (20). However, clinical and pathological association of ANXA1 in AAV remains unclear. In the current study, we measured the plasma levels and renal expression of ANXA1, and further analyzed their association with clinical and pathological parameters in AAV patients.

## Materials and Methods

### Patients and Samples

Thirty-one patients with active AAV receiving renal biopsy diagnosed in Peking University First Hospital from 2016 to 2018 were recruited in the current study. All patients met the Chapel Hill Consensus Conference (CHCC) nomenclature for AAV (21) and had complete clinicopathological data. Patients with secondary vasculitis or coexisting renal diseases, for example, lupus nephritis (LN), IgA nephropathy or anti-glomerular basement membrane disease were excluded. Renal specimens from 10 patients with minimal change disease (MCD) and 9 patients with LN were collected as disease controls. Ten renal tissues from the normal part of nephrectomized kidneys were defined as normal on the basis of light microscopy, immunofluorescence and electron microscopy and then were used as normal controls.

**Figure 1:**
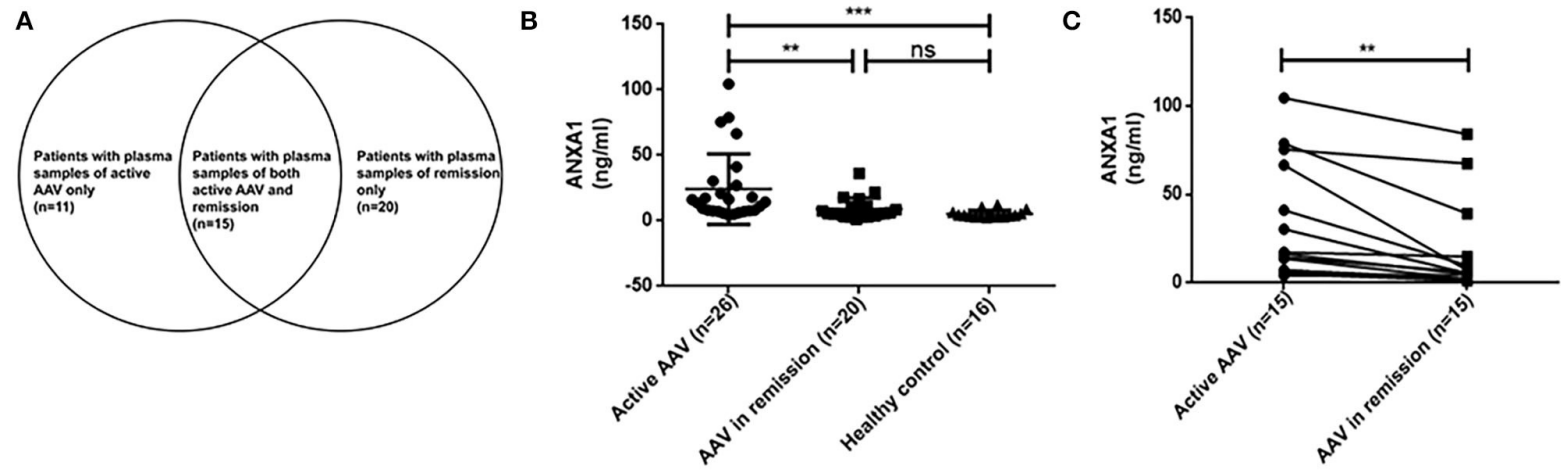
Details of plasma samples of AAV patients **(A)**. Plasma levels of ANXA1 in active AAV patients (*n* = 26) and controls (AAV in remission, *n* = 20; healthy control, *n* = 16) **(B)**. Comparisons of ANXA1 levels in 15 AAV patients with sequential plasma samples (pairs = 15) **(C)**. AAV, anti-neutrophil cytoplasmic antibody (ANCA)-associated vasculitis; ANXA1, annexin A1; ns, no significance (*P* > 0.05). **P < 0.01, ***P < 0.001.

Among the 31 above mentioned active AAV patients, plasma samples were collected in 26 patients for measurement of plasma levels of ANXA1. Plasma samples of 35 patients with AAV in remission were also collected at their regular ambulatory follow-up. Fifteen out of these 35 patients were also among the above mentioned 31 patients who also had plasma samples of active stage of AAV. The details were depicted in Figure 1A. Plasma samples of 16 healthy blood donors were collected as normal controls. These blood samples were centrifuged at 2,000 *g* for 15 min at 4°C within 30 min after collection. Then the plasma samples were stored at −80°C until use. Repeated freeze/thaw cycles were avoided.

The disease activity of AAV patients was assessed according to the Birmingham vasculitis activity score (BVAS) (22). As previously described, remission was defined as “absence of disease activity attributable to active disease qualified by the need for ongoing stable maintenance immunosuppressive therapy” (complete remission), or “at least 50% reduction of disease activity score and absence of new manifestations” (partial remission) (23). The renal response to treatment was evaluated at 6 months after initiation of immunosuppressive therapy, according to the following criteria described previously (24–26): (1) complete recovery of renal function was indicated by normalization of renal function and resolution of hematuria; (2) partial recovery of renal function was indicated by stabilization or improvement of renal function, with serum creatinine (Scr) ≥133 μmol/l but dialysis independent; and (3) treatment failure was indicated by progressive decline in kidney function with persistence of active urinary sediment despite immunosuppressive therapy.

Baseline data at diagnosis as well as follow-up information of patients were collected from electronic medical records of our hospital. Each participant signed the informed consent. This research was conducted in accordance with the Declaration of Helsinki and was approved by the Ethics Committee of Peking University First Hospital.

### Detection of Plasma ANXA1

Plasma ANXA1 was detected by enzyme-linked immunosorbent assay (ELISA) using the commercial kit (ab222868, Abcam, Cambridge, UK). The detection was carried out according to the manufacturer's guidance. In brief, the 96-well plate was pre-coated with polyclonal antibody specific for human ANXA1. The diluted ANXA1 standards and plasma samples were added to appropriate wells. After 2-h incubation at room temperature and five washes, biotinylated antibody was added to each well. Following 1-h incubation and five washes, the streptavidin-peroxidase conjugate was added to each well. Then the plate was incubated at room temperature for another 30 min. After five washes, chromogen substrate was added in each well and then the plate was incubated in ambient light for 15 min. The reaction was stopped by adding stop solution. The absorbance was measured at 450 nm within 15 min. The concentration of ANXA1 in plasma samples was determined in accordance with the standard curve.

### Renal Histopathology

Renal pathology of AAV patients was evaluated according to the standardized protocol described previously (27–29). The glomerular lesions, including crescents, fibrinoid necrosis and glomerulosclerosis, were expressed as the proportion of affected glomeruli in total glomeruli in the specimens. In terms of the area of the tubulointerstitial compartment affected, tubular and interstitial lesions were semi-quantitatively scored as followed: interstitial fibrosis (- for 0%, + for 0–50% and ++ for >50%), interstitial infiltrate (– for 0%, + for 0–20%, ++ for 20%-50% and +++ for >50%) and tubular atrophy (- for 0%, + for 0–50% and ++ for >50%).

### Immunohistochemistry Staining of ANXA1 in Renal Biopsies

Slides with formaldehyde-fixed renal tissues were deparaffinized in xylene and rehydrated in graded ethanol. After washed with phosphate buffer saline (PBS), slides were immersed in 3% hydrogen peroxide for 30 min at room temperature. After another three washes with PBS, antigen retrieval was then performed through heating the slides in Tris-ethylene diamine tetraacetic acid (EDTA) buffer (pH 9.0) in an 800 W microwave oven for 2 min and then at 200 W one for 8 min. Then the slides were cooled to room temperature and washed with PBS, followed by blockage of non-specific staining with 3% bovine serum albumin (BSA) in PBS at 37°C for 1 h. After removing BSA without washing, the primary antibody, rabbit anti-human ANXA1 (1:4000, ab214486; Abcam, Cambridge, UK) was added and then incubated overnight at 4°C. Slides of the negative control were incubated with 3% BSA instead of primary antibody. After 5 washes in PBS, slides were incubated with the secondary antibody (PV9001, ZSGB-Bio, Beijing, China) at room temperature for 20 min. Then the slides were developed in fresh hydrogen peroxide with 3-3'-diaminobenzidine tetrahydrochloride solution for 45 s. Finally, the slides were incubated with haematoxylin for 8 min to attain nuclear staining and then dehydrated by graded alcohol and xylene. For quantitative analyses of immunohistochemical staining, all the glomeruli and at least 10 fields of tubulointerstitium per kidney slide were observed blindly at ×400.

The Image-Pro Plus analysis software (version 6.0; Media Cybernetics, Dallas, TX, USA) was used to assess the renal staining results of ANXA1 quantitatively. The optical intensity threshold was corrected to 0–250 in the process of analyses. Positive signals were shown as the mean optical density (integrated option density/area).

### Co-localization of ANXA1 With Markers of Different Cell Types

To investigate concrete locations of ANXA1 in the kidney of AAV patients, double immunofluorescence of ANXA1 and specific markers of different cell types including renal intrinsic cells as well as inflammatory cells was performed. Glomerular endothelial cells, mesangial cells and podocytes were marked with mouse anti-human CD31 (1:50, sc-376764; Santa Cruz Biotechnology, CA), mouse anti-human integrin-α8 (1:50, sc-365798; Santa Cruz Biotechnology, CA) and mouse anti-human synaptopodin (1:50, MAB8977; R&D Systems, Minneapolis, MN, USA), respectively. Proximal tubular epithelial cells were identified with lotus tetragonolobus lectin (LTL, 1:200, FL-1321; Vector Labs, USA). Distal tubules and collecting ducts were marked with mouse anti-human Calbindin D28K (1:200, sc-365360; Santa Cruz Biotechnology, CA). Neutrophils and monocytes/macrophages were identified with rabbit anti-human neutrophil elastase (NE; 1:50, ab131260; Abcam, Cambridge, UK) and mouse anti-human CD68 (1:50, ab955; Abcam, Cambridge, UK), respectively. CD3+, CD4+ and CD8+ T cells were marked with rat anti-human CD3 (1:250, ab11089; Abcam, Cambridge, UK), rabbit anti-human CD4 (1:50, ab133616; Abcam, Cambridge, UK) and mouse anti-human CD8 (1:50, ab17147; Abcam, Cambridge, UK), respectively. In the co-localization of ANXA1 with glomerular endothelial cells, mesangial cells, podocytes, distal tubules and collecting ducts, monocyte/macrophages and CD8+ T cells, rabbit anti-human ANXA1 (1:100, ab214486; Abcam, Cambridge, UK) was used as primary antibody. In the co-localization of ANXA1 with neutrophils, proximal tubular epithelial cells and CD4+ T cells, mouse anti-human ANXA1 (1:100, sc-12740; Santa Cruz Biotechnology, CA) was used as primary antibody. After deparaffinization, antigen retrieval and blockage of non-specific staining according to the above mentioned methods, the specimens were incubated with mixture of primary antibodies overnight at 4°C. After 5 washes with PBS, the specimens were incubated with secondary antibodies mixture of Alexa Fluor 488 (AF488) -labeled goat anti-rabbit IgG (1:200, A-32731; Invitrogen, USA) and Alexa Fluor 555 (AF555) -labeled goat anti-mouse IgG (1:200, A-32727; Invitrogen, USA) at 37°C for 1 h. Notably, in the co-localization of ANXA1 with proximal tubular epithelial cells, LTL was mixed with secondary antibodies. In the co-localization of ANXA1 and CD3, AF555-labeled goat anti-rat IgG (1:200, 4417; Cell Signaling Technology, USA) was used as secondary antibody. After washed with PBS five times, the specimens were stained with 4,6-diamidino-2-phenylindole (DAPI) and finally mounted with Mowiol. For the negative controls, the mixture of primary antibodies was replaced with 3% BSA. Zeiss LSM 710 confocal microscope (Zeiss, Jena, Germany) and fluorescence microscope (90i, Nikon, Japan) were used to capture confocal images. Then these images were exported from the ZEN 2012 (blue edition) microscopy software.

### Statistical Analysis

Data were expressed as median and interquartile range (IQR; for data that were in skewed distribution) or mean ± SD (for data that were normally distributed) for continuous variables, or number (%) for qualitative variables. Differences between groups were analyzed by *t*-test for normally-distributed data or by non-parametric test for non-normally distributed data. Pearson's correlation or Spearman's rank correlation was performed to assess the correlation between two variables as appropriate. *P* < 0.05 was considered statistically significant. Data analyses were performed with SPSS 22.0 (IBM Corp., Armonk, NY).

## Results

### General Data of the Patients

Among the 31 patients with active AAV, 13 (41.9%) were male and 18 (58.1%) were female, with a median age of 65 (IQR 60–69) years at diagnosis. All the patients were MPO-ANCA positive. The median level of initial serum creatinine was 253.5 (IQR 195.3–371.0) μmol/L. The median BVAS of these 31 patients was 15 (IQR 15–20). Among the 35 AAV patients in remission, the median serum creatinine level was 147.0 (IQR 108.0–186.0) μmol/L, and the BVAS was 0 for each of these patients. General data of the patients were listed in Table 1.

**Table 1:** General data of patients with AAV.

**Parameters**	**Active stage**	**Remission stage**
**Demographic data**		
Subjects, *n*	31	35
Male/female	13/18	18/17
Age at diagnosis, median (IQR)	65 (60–69)	
**Clinical manifestation**		
Skin rash, *n* (%)	4 (12.9)	
Arthralgia, *n* (%)	1 (3.2)	
Muscle pain, *n* (%)	6 (19.4)	
Pulmonary, *n* (%)	15 (48.4)	
ENT, *n* (%)	7 (22.6)	
Ophthalmic, *n* (%)	2 (6)	
Gastrointestinal, *n* (%)	0 (0)	
Nervous system, *n* (%)	4 (12.9)	
**BVAS**		
Median (IQR)	15 (15–20)	0(0-0)
Range	12–29	0-0
**Laboratory data**		
MPO-ANCA (%)	31 (100)	
**Serum creatinine**, **μmol/L**		
Median (IQR)	253.5 (195.3–371.0)	147.0 (108.0–186.0)
Range	59.4–921.5	63.0–823.0
**Urinary protein (g/24h)**		
Median (IQR)	1.03 (0.36–2.86)	
Range	0.02–4.71	
Hematuria (%)	30 (96.8)	
**Pathological data**		
**Total glomeruli**		
Median (IQR)	28 (20–40)	
Range	15–76	
**Glomerular lesions (%)**		
Total crescents	48.9 ± 20.0	
Cellular crescents	33.6 ± 17.0	
Fibrous crescents	12.0 (0.0–22.5)	
Glomerulosclerosis	7.4 (4.4–22.5)	
**Tubulointerstitial lesions**, ***n***		
Interstitial infiltration (-/+/++/+++)	0/5/19/7	
Interstitial fibrosis (-/+/++)	0/23/8	
Tubular atrophy (-/+/++)	1/24/6	

*BVAS, Birmingham Vasculitis Activity Score; ENT, ear, nose and throat*.

Among the 31 AAV patients, only three patients did not receive any immunosuppressive treatment before renal biopsy. Twenty-eight patients received oral glucocorticoids before renal biopsy. No patients received high-dose methylprednisolone pulse therapy, plasma exchange, cyclophosphamide or rituximab before renal biopsy. Among the 10 MCD patients, no patient received glucocorticoids treatment before renal biopsy. Among the 9 LN patients, 3 patients received oral glucocorticoids treatment before renal biopsy.

### Plasma Levels of ANXA1

As above mentioned, we tested plasma samples from 26 out of the above mentioned 31 patients with active AAV, 35 AAV patients in remission, including 15 paired samples of both active stage and remission stage of AAV, as well as 16 healthy blood donors. The plasma levels of ANXA1 in active AAV patients were significantly higher than those of AAV patients in remission as well as those of healthy controls [14.1 (IQR 7.6–27.9) vs. 5.6 (IQR 3.6–11.3] ng/ml, *P* = 0.003; 14.1 (IQR 7.6–27.9) vs. 4.1 (IQR 3.1–6.4) ng/ml, *P* < 0.001, respectively) (Figure 1B).

**Figure 2:**
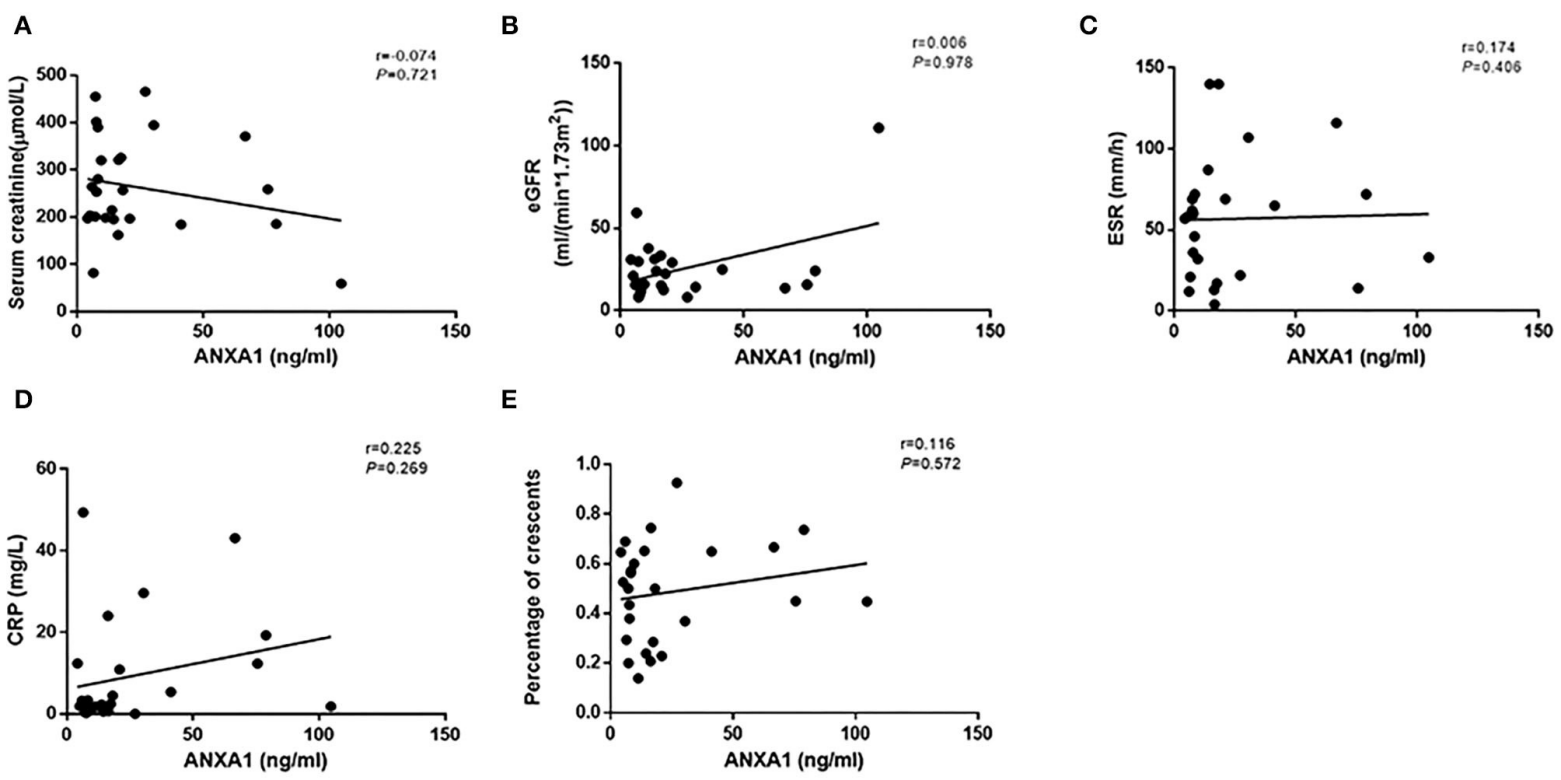
Correlation analyses of ANXA1 levels in plasma of active AAV patients and serum creatinine **(A)**, eGFR **(B)**, ESR **(C)**, CRP **(D)** and the proportion of crescents **(E)**, respectively. ANXA1, annexin A1; eGFR, estimated glomerular filtration rate; ESR, erythrocyte sedimentation rate; CRP, C-reactive protein.

Then we compared plasma levels of ANXA1 in 15 AAV patients with sequential plasma samples of both active stage and remission. The plasma levels of ANXA1 were significantly higher in patients with active AAV than in their remission stage [16.4 (IQR 7.2–66.6) vs. 5.5 (IQR 2.2–14.9) ng/ml, *P* = 0.001], which was consistent with the above mentioned results. In detail, there were 14 patients in active stage with higher plasma levels of ANXA1 than those in remission, while there was only one patient exhibiting a slight increase in the plasma in remission compared with that in active stage (Figure 1C). However, correlation analyses did not show any significant correlation between plasma levels of ANXA1 with clinicopathological parameters in AAV patients. The details were shown in Figure 2.

### Immunohistochemistry Analysis of ANXA1 in Renal Biopsies

**Figure 3:**
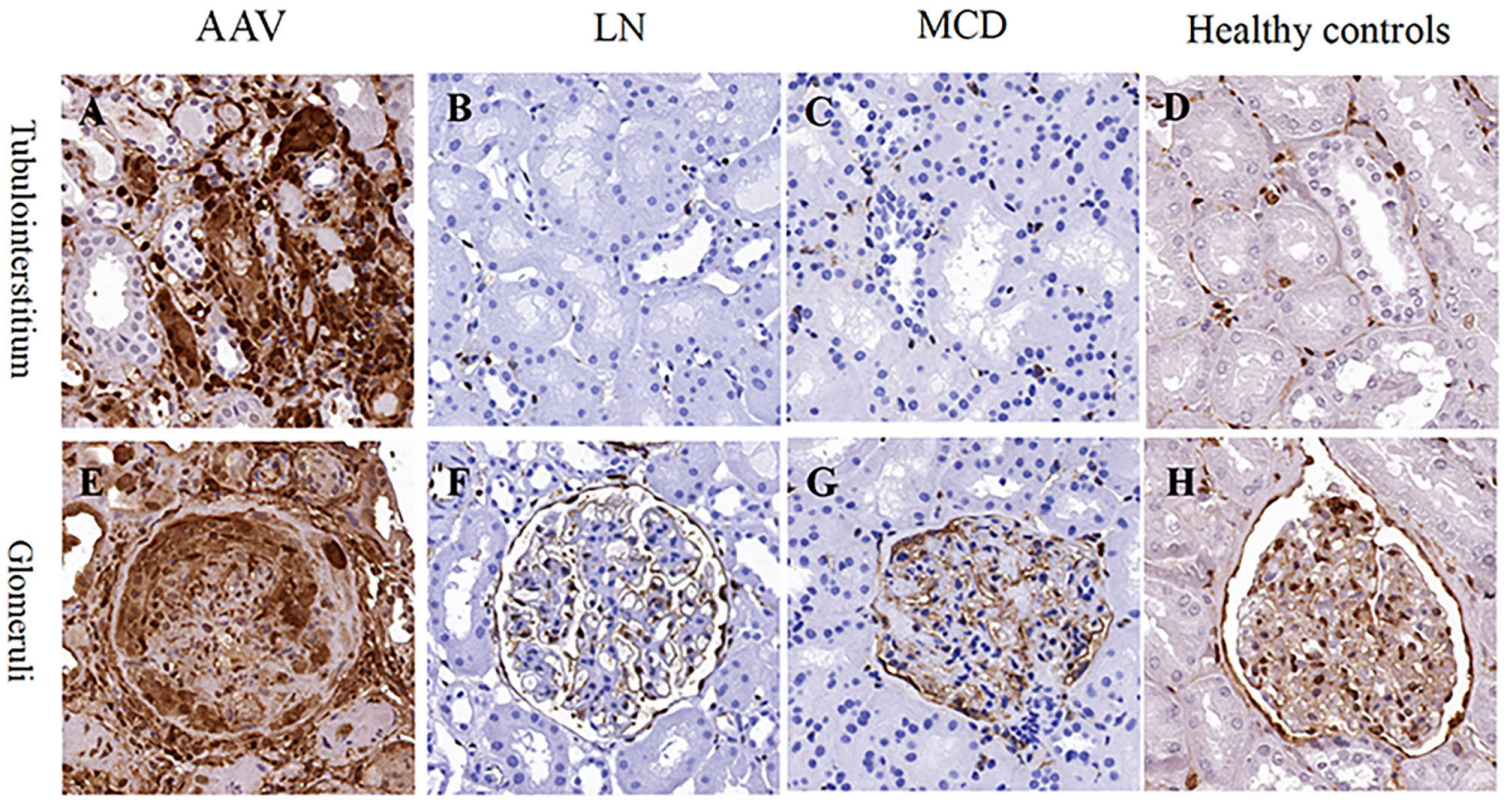
Immunohistochemistry staining for ANXA1 in tubulointerstitium **(A–D)** and glomeruli **(E–H)** in patients with AAV **(A,E)**, LN **(B,F)** and MCD **(C,G)** as well as healthy controls **(D,H)**, respectively. AAV, anti-neutrophil cytoplasmic antibody (ANCA)-associated vasculitis; ANXA1, annexin A1; LN, lupus nephritis; MCD, minimal change disease.

**Figure 4:**
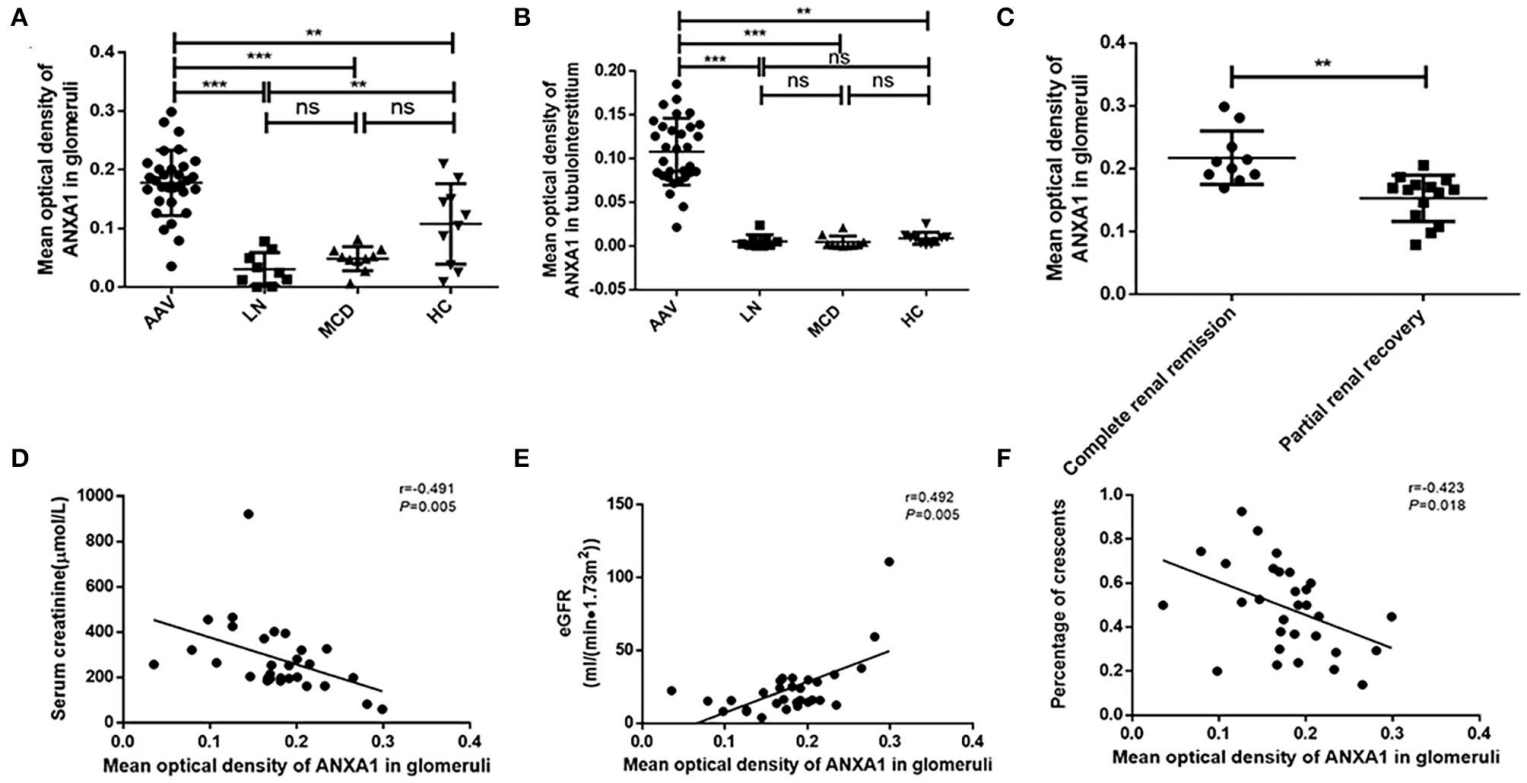
**(A)** Mean optical density of ANXA1 in glomeruli of AAV patients, LN, MCD and healthy controls respectively. **(B)** Mean optical density of ANXA1 in tubulointerstitium of AAV patients, LN, MCD and healthy controls respectively. **(C)** Comparison of ANXA1 expression of patients achieving complete renal recovery with those achieving partial renal recovery. **(D–F)** Correlation analyses of ANXA1 expression in glomeruli of AAV patients with serum creatinine **(D)**, eGFR **(E)** and the proportion of crescents **(F)**, respectively. AAV, anti-neutrophil cytoplasmic antibody (ANCA)-associated vasculitis; ANXA1, annexin A1; eGFR, estimated glomerular filtration rate; HC, healthy control; LN, lupus nephritis; MCD, minimal change disease; ns, no significance (*P* > 0.05). **P < 0.01, ***P < 0.001.

Immunohistochemistry of renal specimens were performed to evaluate the renal expression of ANXA1 in 31 AAV patients in active stage. The results demonstrated that ANXA1 was widely expressed in tubulointerstitium and glomeruli (Figure 3). Compared with normal controls, the mean optical densities of ANXA1 in both glomeruli and tubulointerstitium of AAV patients were significantly higher (0.178 ± 0.056 vs. 0.108 ± 0.068, *P* = 0.002; 0.108 ± 0.038 vs. 0.009 ± 0.007, *P* = 0.001, respectively) (Figures 4A,B). Moreover, patients with LN and MCD exhibited significantly weaker expression of ANXA1 in glomeruli and tubulointerstitium than AAV patients (0.031 ± 0.028 vs. 0.178 ± 0.056, *P* < 0.001; 0.049 ± 0.021 vs. 0.178 ± 0.056, *P* < 0.001; 0.002 (IQR 0.001–0.008) vs. 0.111 (IQR 0.081–0.137), *P* < 0.001; 0.002 (IQR 0.001–0.007) vs. 0.111 (IQR 0.081–0.137), *P* < 0.001, respectively) (Figures 4A,B).

### Co-localization of ANXA1 With Various Cell Types

**Figure 5:**
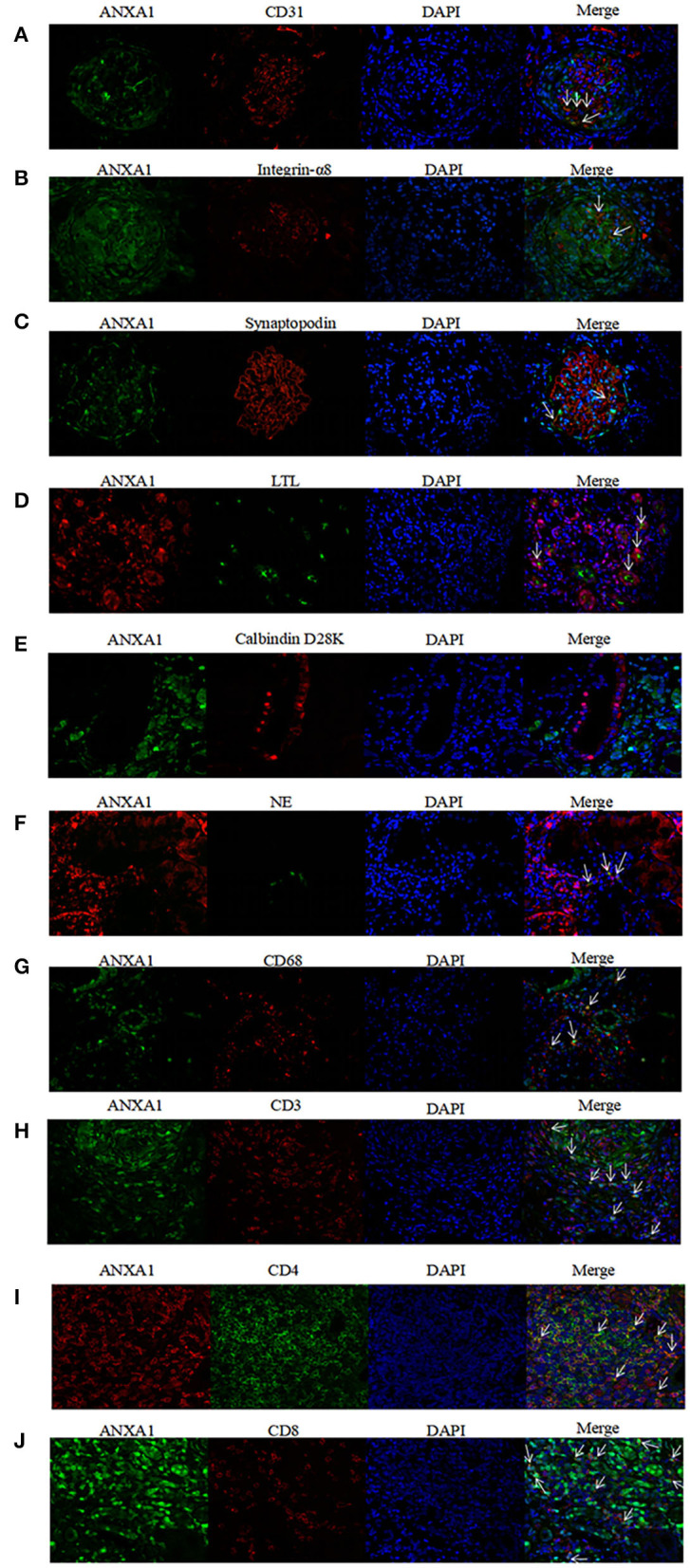
Co-localization of ANXA1 and CD31 **(A)**, integrin-α8 **(B)**, synaptopodin **(C)**, LTL **(D)**, Calbindin D28K **(E)** and NE **(F)**, CD68 **(G)**, CD3 **(H)**, CD4 **(I)** and CD8 **(J)** in renal specimens of AAV patients respectively. AAV, anti-neutrophil cytoplasmic antibody (ANCA)-associated vasculitis; ANXA1, annexin A1; CD3, cluster of differentiation 3; CD4, cluster of differentiation 4; CD8, cluster of differentiation 8; CD31, cluster of differentiation 31; CD68, cluster of differentiation 68; LTL, lotus tetragonolobus lectin; NE, neutrophil elastase.

**Figure 6:**
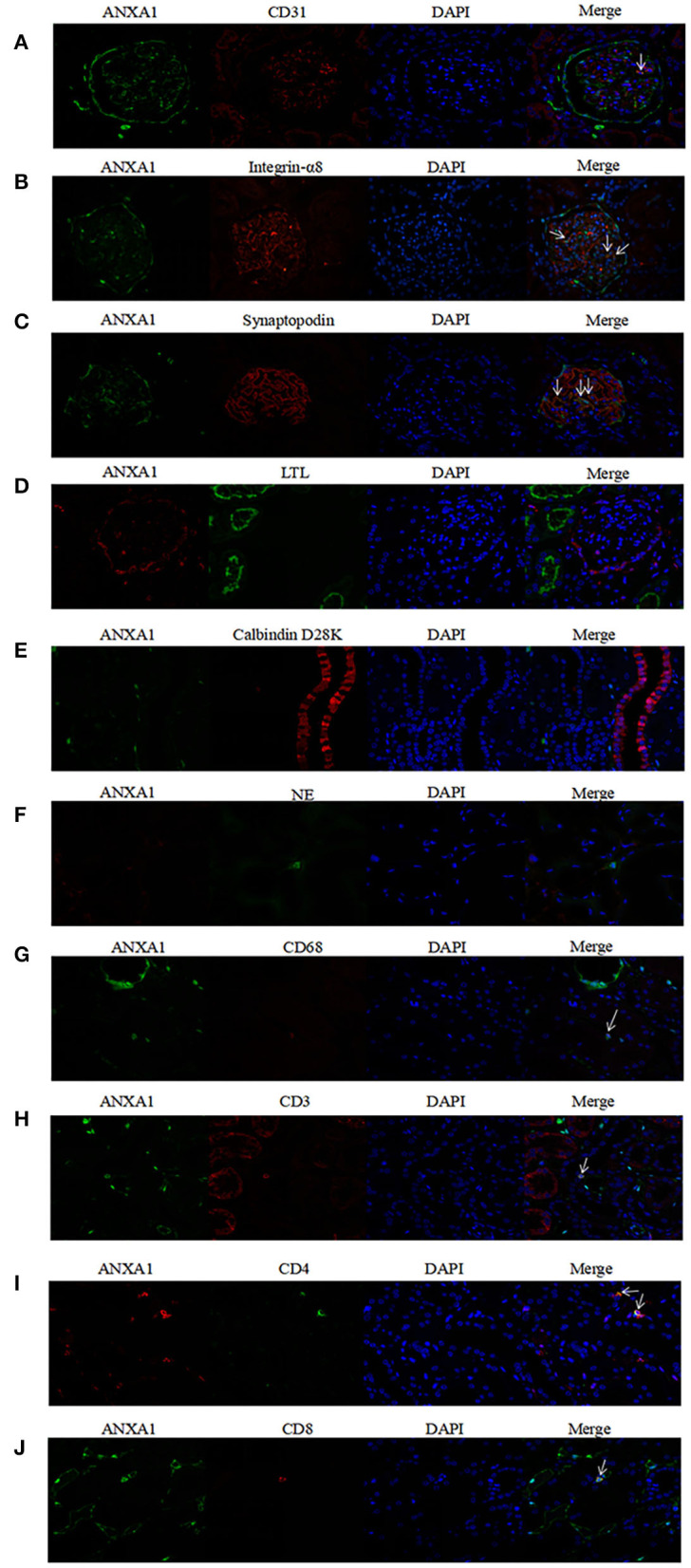
Co-localization of ANXA1 and CD31 **(A)**, integrin-α8 **(B)**, synaptopodin **(C)**, LTL **(D)**, Calbindin D28K **(E)** and NE **(F)**, CD68 **(G)**, CD3 **(H)**, CD4 **(I)** and CD8 **(J)** in renal specimens of healthy controls, respectively. AAV, anti-neutrophil cytoplasmic antibody (ANCA)-associated vasculitis; ANXA1, annexin A1; CD3, cluster of differentiation 3; CD4, cluster of differentiation 4; CD8, cluster of differentiation 8; CD31, cluster of differentiation 31; CD68, cluster of differentiation 68; LTL, lotus tetragonolobus lectin; NE, neutrophil elastase.

According to the results of immunohistochemical assay, we found that ANXA1 was highly expressed in glomeruli and tubulointerstitium of AAV patients. Therefore, the double immunofluorescence staining for ANXA1 and markers of various cell types was performed to further investigate the specific cell types with increased ANXA1 expression. The partial merge of ANXA1 with CD31, integrin-α8, synaptopodin, LTL, NE and CD68 was observed in renal specimens of AAV patients, which suggested that ANXA1 expressed in glomerular endothelial cells, mesangial cells, podocytes, proximal tubular epithelial cells, neutrophils and monocytes/macrophages (Figures 5A–D,F,G). ANXA1 was expressed by CD3+, CD4+ and CD8+ T cells in both AAV patients and healthy controls (Figures 5H–J, 6H–J). The partial merge of ANXA1 with glomerular endothelial cells, mesangial cells, podocytes and monocytes/macrophages was also observed in healthy controls (Figures 6A–C,G). The merge of ANXA1 with NE and LTL was not observed in healthy controls (Figures 6D,F). No obvious co-localization of ANXA1 with distal tubules or collecting ducts was observed in AAV patients or healthy controls (Figures 5E, 6E).

### Associations Between Renal ANXA1 Expression and Clinicopathological Parameters of Patients With Active AAV

We further investigated associations between ANXA1 expression in renal specimens and clinicopathological parameters of AAV patients. The correlation analyses among the 31 AAV patients showed that the mean optical density of ANXA1 in glomeruli correlated with the levels of serum creatinine and eGFR at renal biopsy (*r* = −0.491, *P* = 0.005; *r* = 0.492, *P* = 0.005, respectively) (Figures 4D,E). Besides, the expression of ANXA1 in glomeruli correlated with the proportion of crescents (*r* = −0.423, *P* = 0.018) (Figure 4F). Among the 25 active AAV patients with follow-up data, complete renal recovery occurred in 10 patients (40%), with 14 patients (56%) achieving partial renal recovery and 1 patient (4%) experiencing treatment failure. The baseline expression of ANXA1 in glomeruli of patients achieving complete renal recovery was significantly higher than those achieving partial renal recovery [0.206 (IQR 0.189–0.246) vs. 0.167 (IQR 0.122–0.176), *P* = 0.001] (Figure 4C). The levels of ANXA1 were significantly different among AAV patients with various degree of interstitial infiltration (*P* = 0.010), i.e., patients with severer interstitial infiltration had higher levels of ANXA1. No significant correlation was observed between the levels of ANXA1 in tubulointerstitium and interstitial fibrosis or tubular atrophy (*P* = 0.176 and *P* = 0.846, respectively).

## Discussion

ANXA1 is a 37-kDa protein and a member of annexin superfamily binding phospholipid dependent on calcium (30). ANXA1 is also a glucocorticoid-regulated protein and has anti-inflammatory and pro-resolving effects, including blockage of leukocytes migration and acceleration of neutrophils apoptosis (31). ANXA1 inhibits neutrophils adhesion and recruitment and therefore promotes resolution of inflammation (13). ANXA1 mimetic peptide (Ac2-26) augments neutrophils apoptosis (15). ANXA1 promotes macrophages skewing toward M2 phenotype, which contributes to the resolution of inflammation and tissue repair (32). In addition, ANXA1 shows protective roles in various renal diseases. In mice with diabetic nephropathy, ANXA1 ameliorates kidney injuries through facilitating the resolution of inflammation (33). ANXA1 mimetic peptide (Ac2-26) protects against renal ischemia/reperfusion injury by abortion of neutrophil extravasation and attenuation of macrophage infiltration (34).

Our previous study found that ANXA1 was upregulated in kidney of patients with diabetic nephropathy and was correlated with renal function and renal outcomes (33). Ka et al. (35) demonstrated that urinary levels of ANXA1 were significant higher in secondary glomerular diseases (such as diabetic nephropathy and LN) than in primary glomerular disorders and normal controls. However, the significance of ANXA1 in AAV remains unknown. Thus, the present study investigated the association between ANXA1 with clinical and pathological parameters of AAV.

In the current study, we found that compared with healthy controls, the levels of ANXA1 were upregulated in kidneys of patients with active AAV and were associated with renal disease severity of AAV, including eGFR, serum creatinine and proportion of crescent formation, and to some extent, interstitial infiltration, in renal specimens. Moreover, the levels of renal expression of ANXA1 were associated with renal response to treatment. Plasma levels of ANXA1 were significantly higher in active AAV patients than in AAV patients in remission as well as healthy controls. However, further assessment did not show any significant correlation between ANXA1 levels in plasma and clinicopathological parameters of AAV. We speculated that in the context of active AAV, circulating ANXA1 translocated to local sites of inflammation, e.g., kidneys, and exerted its pro-resolving effects. As mentioned above, ANXA1 could act on various inflammatory cells and further promote resolution of inflammation, such as inhibition of neutrophil and macrophage infiltration, augmentation of neutrophil apoptosis and skewing of macrophage toward M2 phenotype, which might explain the association of ANXA1 with disease severity in AAV. Besides, ANXA1 could promote mucosal wound repair, muscle injury regeneration and cardiac repair (32, 36, 37), which might explain the association of renal ANXA1 expression with renal responses to treatment. In addition, we found that ANXA1 was expressed by neutrophils, monocytes/macrophages, T cells, mesangial cells and epithelial cells, which were consistent with the results of previous studies (38–40).

There are some limitations of the current study. First, the sample size was relatively small, and all the patients recruited were MPO-ANCA positive, since MPO rather than PR3 is the main target antigen of ANCA in China (4, 41). Second, glucocorticoids may affect the expression of ANXA1, but a majority of AAV patients recruited in the current received glucocorticoids before renal biopsy. In clinical practice, physicians usually give patients immunosuppressive therapy once the diagnosis of AAV established, even before renal biopsy. Therefore, it is very difficult to get renal specimens of AAV patients before using glucocorticoids. Third, since this was a retrospective study, all the samples were obtained from our biobank, and we did not have the samples of peripheral blood mononuclear cells (PBMCs) of these patients in our biobank. Therefore, we are not able to measure ANXA1 expression in PBMCs of these patients.

In conclusion, the renal expression of ANXA1 was upregulated in AAV patients and was correlated with the severity of renal injury.

## Data Availability Statement

The original contributions presented in the study are included in the article, further inquiries can be directed to the corresponding author.

## Ethics Statement

The studies involving human participants were reviewed and approved by Ethics Committee of Peking University First Hospital. The patients/participants provided their written informed consent to participate in this study.

## Author Contributions

R-XW finished the whole experiment, analyzed statistics, and drafted the manuscript. LW, S-FC, Z-YL, MC, and M-HZ designed the study, participated interpretation of data, and revised the manuscript. Z-YL had full access to all of the data, provided final approval of the submitted manuscript, guarantor of this work, and took responsibility for the integrity of the data and the accuracy of the data analysis. All authors read and approved the manuscript.

## Funding

This study was supported by National High Level Hospital Clinical Research Funding (Multi-center Clinical Research Project of Peking University First Hospital, No. 2022CR52), Capital's Funds for Health Improvement and Research [No. 2020-2-4073], Grants from four the National Natural Science Funds (Nos. 81870477, 81870478, 81900640, and 82090020/82090021), and CAMS Innovation Fund for Medical Sciences (2019-I2M-5-046).

## Conflict of Interest

The authors declare that the research was conducted in the absence of any commercial or financial relationships that could be construed as a potential conflict of interest.

## Publisher's Note

All claims expressed in this article are solely those of the authors and do not necessarily represent those of their affiliated organizations, or those of the publisher, the editors and the reviewers. Any product that may be evaluated in this article, or claim that may be made by its manufacturer, is not guaranteed or endorsed by the publisher.
